# Urinary pyrethroid metabolite and hearing threshold shifts of adults in the United States: A cross-sectional study

**DOI:** 10.1371/journal.pone.0275775

**Published:** 2022-10-17

**Authors:** Lili Long, Yuedi Tang

**Affiliations:** 1 Department of Otorhinolaryngology, Sichuan University Hospital of Sichuan University, Chengdu, Sichuan, China; 2 Department of Otorhinolaryngology Head & Neck Surgery, West China Hospital of Sichuan University, Chengdu, Sichuan, China; King Faisal Specialist Hospital and Research Center, SAUDI ARABIA

## Abstract

Hearing loss (HL) is a global health problem with a high prevalence and profound socioeconomic impact. Pyrethroids are one of the most commonly used insecticides. Although previous studies have reported the relationship between pyrethroids and neurotoxicity, little is known about the effect of pyrethroid exposure on the auditory system among the general population. This study is aimed to investigate the association of pyrethroid exposure with hearing threshold shifts of adults in the United States. A total of 726 adults, aged from 20 to 69 years from the 2011–2012 National Health and Nutrition Examination Survey (NHANES) data were included in the study. Urinary 3-phenoxybenzoic acid (3-PBA), a general pyrethroid metabolite, was used as a biomarker for pyrethroid exposure. HL was defined as a pure-tone average (PTA) at 0.5, 1, 2, 4 kHz ≥ 20 dB in the better ear. Analyses by using multivariate linear regressions were conducted to explore the associations of urinary 3-PBA with PTA hearing threshold shifts. There were no statistically significant correlations between Ln-transformed 3-PBA and either low-frequency or high-frequency hearing thresholds after adjusting for age, gender, race/ethnicity, education level, firearm noise exposure, occupational noise exposure, recreational noise exposure, serum cotinine, BMI, hypertension, and diabetes. However, associations of 3-PBA with both low-frequency and high-frequency hearing thresholds depended on age (*P*_interaction_ < 0.0396 and 0.0017, respectively). Positive associations between Ln-transformed 3-PBA and both low-frequency and high-frequency hearing thresholds were observed in participants aged 20–39 years after adjusting confounders (β = 1.53, 95% CI: 0.04–3.01, and β = 3.14, 95% CI: 0.99–5.29, respectively) with the highest tertile (≥ 0.884 μg/g creatinine) of 3-PBA compared with the lowest tertile (< 0.407 μg/g creatinine). The possibility of interaction between 3-PBA and age on the hearing threshold shifts indicated that pyrethroid insecticides were prone to be more toxic to auditory system in younger adults than in older ones. Further studies will be required to confirm these findings.

## Introduction

Hearing loss (HL) is the most common human sensory deficit. It is estimated that over five percent of the world’s population (~360 million people) are currently living with a disabling HL [1]. HL is associated with dementia, depression and social isolation, adversely affects educational attainment, and impairs the quality of life [2–5]. The estimated HL-related direct medical costs and indirect costs have reached from $3.3 million to $12.8 million per year in the United States [6]. HL-related unemployment costs the UK economy £24.8 billion per year [1]. This public health problem is escalating. Predictions suggest that 630 million people worldwide could have disabling HL by 2030; this number may reach nearly 900 million by 2050 unless appropriate action is taken [7]. This increasing prevalence and high societal burden, makes prevention and intervention of HL a medical and public health priority, and studying risk factors is essential to developing preventive and therapeutic strategies. Besides common causes of hearing impairment, like noise exposure and aging factors, links between exposure to environmental pollutants and the development of hearing impairment is a topic of growing concern [8].

Pyrethroid insecticides are synthetic chemicals derived from natural pyrethrins extracted from the flowers of Chrysanthemum, and they are more stable in the sunlight than pyrethrin. They are the second generation of insecticides which were developed mostly during World War II [9]. Due to high insecticidal effectiveness, low acute toxicity to mammals, pyrethroids are used widespread and increasing in agriculture, as well as in homes, schools, parks, apartments, and work settings to kill insects [10]. They are also found in consumer products, including pet sprays and shampoos, head lice, flea and tick treatments, and mosquito repellents [10].

More people, including children, are exposed to pyrethroids as the consumption of these synthetic chemicals increases. Previous studies have suggested associations between exposure to pyrethroids and various neurotoxic impairment in humans such as numbness, dizziness, motor neuron disorder, and memory impairment [11–13]. Though it has been proposed that pyrethroids might affect the auditory system and lead to hearing impairment, studies investigating the relationship between pyrethroid exposure and hearing impairment are few and contradictory [14–19]. Only one previous study has investigated the effects of pyrethroid exposure on hearing impairment in adolescents in the general population without occupational exposure [15]. Therefore, we explored the association between pyrethroid exposure and hearing threshold shifts of adults in the United States who participated in a nationally representative survey, the National Health and Nutrition Examination Survey (NHANES, 2011–2012).

## Methods

### Ethics statement

All NHANES data for analysis in this study were publicly available on the NHANES website (https://wwwn.cdc.gov/nchs/nhanes/continuousnhanes/default.aspx?BeginYear=2011). The protocols for NHANES 2011–2012 were approved by the National Center for Health Statistics (NCHS) of the Centers for Disease Control and Prevention (CDC) Institutional Review Board in accordance with the provisions of the Declaration of Helsinki. Written informed consents were obtained from all subjects.

### Study population

**Fig 1:**
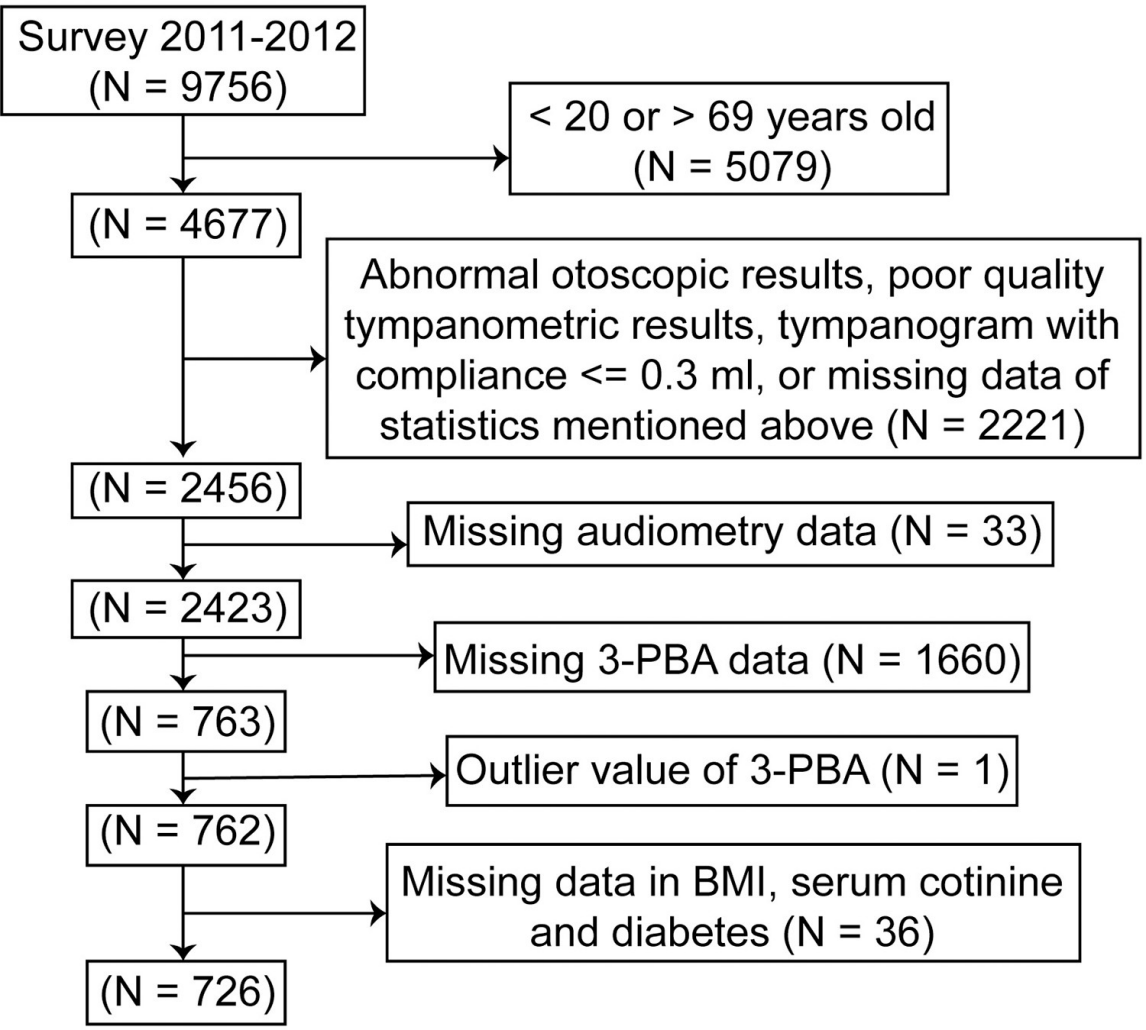
Flow chart of the selection process. NHANES, National Health and Nutrition Examination Survey.

NHANES is a national survey conducted every year by the NCHS of CDC using a complex, multistage, probability study design to be representative of the noninstitutionalized U.S. civilian population. The survey includes a series of person interviews, physical examinations, and laboratory testing data. The current study data was collected from the 2011–2012 cycle of NHANES, the only cycle where both audiometry and urinary pyrethroid metabolites data were available for subjects aged 20–69 years. 726 adults were finally included in the study. All steps of the selection procedure are shown in Fig 1.

### Urinary pyrethroid metabolites measurement

Urinary concentrations of pyrethroid metabolites, including 3-phenoxybenzoic acid (3-PBA), 4-fluoro-3-phenoxybenzoic acid (4F-PBA) and trans-3-(2,2-dichlorovinyl)-2,2-dimethylcylopropane carboxylic acid (trans-DCCA) were measured using high-performance liquid chromatography with a gradient elution program. Sensitive detection of the analytes is performed by a triple quadrupole mass spectrometer with a heated electrospray ionization source. The approach was based on a modification of the method of Beeson, et al. and Olsson et al. [20,21]. More details regarding the laboratory analytical methods and laboratory quantification procedure are available on the website (https://wwwn.cdc.gov/Nchs/Nhanes/2011-2012/UPHOPM_G.htm).

The detection rate of 3-PBA was about 92.1%, whereas only a small percentage of the other two metabolites was observed above the limit of detection (LOD) (Table 1). Therefore, urinary 3-PBA was considered as a biomarker for pyrethroid exposure in the final analysis. The concentrations below LOD were replaced by LOD divided by the square root of two. We corrected for urinary 3-PBA concentrations by using urinary creatinine concentrations and calculated creatinine-standardized 3-PBA concentrations by dividing individual urinary 3-PBA concentrations by creatinine concentrations. The creatinine concentrations were measured using a Roche/Hitachi Modular P Chemistry Analyzer (https://wwwn.cdc.gov/Nchs/Nhanes/2011-2012/ALB_CR_G.htm).

**Table 1:** Detection rates of urine pyrethroid metabolites among participants, NHANES 2011–2012.

Metabolites	Limit of detection (μg/L)	Detection rate (%)
3-PBA	0.1	92.1
4F-PBA	0.1	15.4
Trans-DCCA	0.6	8.2

Abbreviations: 3-PBA, 3-phenoxybenzoic acid; 4F-PBA, 4-fluoro-3-phenoxy-benzoic acid; trans-DCCA, trans-3-(2,2-dichlorovinyl)-2,2-dimethylcylopropane carboxylic acid.

### Audiometric measurement

All audiometric measurements were conducted in a dedicated sound-isolating booth by a trained technician. Hearing thresholds were obtained for both ears of the subjects at frequencies between 500 and 8000 Hz. The detailed audiometry procedure and analytical methods can be found on the NHANES website (https://wwwn.cdc.gov/Nchs/Nhanes/2011-2012/AUX_G.htm). Pure-tone air condition average (PTA) hearing thresholds were calculated at low-frequency (0.5, 1, and 2 kHz) and high-frequency (4, 6, and 8 kHz) in the better ear. HL was defined as PTA across 0.5, 1, 2 and 4 kHz ≥ 20 dB in the better ear [22].

### Covariates

Potential covariates were collected in the analyses included age, gender, race/ethnicity, education level, noise exposures (i. e. firearm, occupational, and recreational noise), serum cotinine, body mass index (BMI), hypertension, and diabetes. Information on age, gender, race/ethnicity, education level, noise exposures, hypertension, and diabetes were obtained from self-reported questionnaires. BMI data were calculated from weight and height data collected during the physical examination.

### Statistical analysis

The study used Environmental Subsample C Weights (WTSC2YR) of 2011–2012 NHANES cycle to estimate representative measures for the United States general population, following the analytic guidelines of NCHS [23,24]. Urinary 3-PBA levels were Ln-transformed, and distributed into tertiles. Weighted statistical differences in demographic and potential hearing-related covariables between samples grouped by tertiles of 3-PBA were evaluated. Categorical data were shown as numbers and percentages and continuous data were expressed as mean ± standard deviation (SD). Multivariate linear regression analysis was used to test for associations, as regression coefficients (β) and 95% confidence intervals (CIs), between 3-PBA and hearing threshold shifts, adjusting for potential confounders, including age, gender, race/ethnicity, education level, noise exposures, serum cotinine, BMI, hypertension, and diabetes. Tests for a linear trend across tertiles of 3-PBA were performed using the median 3-PBA from each tertile. The interactions of age and gender with 3-PBA in influencing hearing thresholds were also evaluated. Multivariate analysis (linear regression model) stratified by age were performed. All statistical tests were conducted with the R language (version 3.6.1) using EmpowerStats software (X&Y Solutions, Inc.). A *P* value of less than 0.05 was considered to be statistically significant.

## Results

### Characteristics of participants

The study sample comprised 726 adults (age range: 20–69; weighted mean age: 42.81 ± 13.62 years; weighted 52.77% females). The means ± SD of low-frequency and high-frequency PTA hearing thresholds in participants were 7.62 ± 7.43 and 18.53 ± 15.26 dB, respectively. Overall, 11.15% adults had HL. Table 2 shows the weighted characteristics of the study participants in total and by weighted tertiles of urinary 3-PBA levels. The ranges of urinary 3-PBA tertiles 1–3 were <0.407, 0.407–0.884 and ≥ 0.884 μg/g creatinine, respectively. Compared with the participants with the lowest tertile of urinary 3-PBA levels, participants with the highest tertile of urinary 3PBA levels were mostly females, older, had lower educational level, had lower chance of firearm noise exposure, and had worse low-frequency PTAs (all P < 0.05). No statistical differences were observed among different urinary 3-PBA tertile groups in BMI, race/ethnicity, hypertension, diabetes, serum cotinine, chances of exposure to occupational or recreational noise, and the prevalence of HL (all P > 0.05).

**Table 2:** The weighted demographic characteristics of study participants.

Characteristics of study participants	Overall (N ^a^ = 726)	Tertiles of Urinary 3-PBA (μg/g creatinine)			*P* value ^b^
		Tertile 1	Tertile 2	Tertile 3	
		(N ^a^ = 242)	(N ^a^ = 242)	(N ^a^ = 242)	
**Continuous variables, mean ± SD**					* *
Age (years)	42.81 ± 13.62	41.54 ± 13.99	41.31 ± 12.94	45.47 ± 13.42	**0.0007**
BMI (kg/m^2^)	28.78 ± 6.14	28.76 ± 5.64	29.00 ± 6.70	28.60 ± 6.11	0.7852
Low-frequency PTA (dB) ^c^	7.62 ± 7.43	6.59 ± 7.59	7.70 ± 7.18	8.61 ± 7.34	0.0092
High-frequency PTA (dB) ^c^	18.53 ± 15.26	17.30 ± 16.25	18.36 ± 16.18	19.95 ± 13.05	0.1460
**Categorical variables, %**					
Gender (female)	52.77	43.99	51.69	62.80	**0.0001**
**Race/Ethnicity**					0.1925
Mexican American	8.20	7.10	10.20	7.52	
Non-Hispanic White	66.90	65.86	61.37	72.97	
Non-Hispanic Black	10.72	11.17	12.69	8.48	
Other races	14.18	15.88	15.74	11.03	
**Education level**					**<0.0001**
Below high school	14.72	7.88	18.70	18.21	
High school	18.23	13.04	19.19	22.73	
Above high school	67.05	79.08	62.12	59.06	
Hypertension	26.21	25.99	26.77	25.94	0.9746
Diabetes	7.06	4.90	7.39	9.00	0.1948
Serum cotinine (≥10 ng/ml)	26.33	22.82	31.34	25.45	0.0999
Firearm noise exposure	41.94	41.98	50.86	33.86	**0.0010**
Occupational noise exposure	32.26	30.28	36.49	30.50	0.2681
Recreational noise exposure	13.41	14.06	13.67	12.50	0.8674
Hearing loss ^d^	11.15	10.24	10.90	12.31	0.7553

Abbreviations: BMI, body mass index; PTA, pure-tone average.

^a^ Unweighted sample number in the dataset.

^b^ *P* values of continuous variables and categorical variables were calculated by weighted linear regression model and weighted chi-square test, respectively.

^c^ Low-frequency and high-frequency PTA values in the better ear were computed from the average of hearing thresholds of 0.5, 1 and 2 kHz, 4, 6 and 8 kHz, respectively.

^d^ Hearing loss was defined as PTA at 0.5, 1, 2 and 4 kHz ≥ 20 dB in the better ear.

### Multivariate regression analysis: Association of 3-PBA with hearing thresholds

Table 3 shows associations of urinary 3-PBA levels with low-frequency and high-frequency hearing thresholds using multivariate linear regression model. Ln-transformed 3-PBA levels were converted from a continuous variable to a categorical variable (tertiles), and were used as a continuous term to calculate the linear trend. In the unadjusted model (crude model) and the partially adjusted model (model 1, adjusted for age and gender), the *P* value for trend shows that 3-PBA levels were positively associated with low-frequency PTA hearing threshold shifts. In the fully adjusted model (model 2), significant *P* for trend was not observed among tertiles of 3-PBA levels and hearing threshold shifts (all *P* ≥ 0.05).

**Table 3:** Multivariable linear regression models for outcome of hearing thresholds.

Urinary 3-PBA	β (95% CI), *P* value of low-frequency PTA (dB)			β (95% CI), *P* value of high-frequency PTA (dB)		
(μg/g creatinine)	Crude Model	Model 1	Model 2	Crude Model	Model 1	Model 2
Continuous	0.88 (-0.05, 1.81)	0.28 (-0.57, 1.13)	0.18 (-0.65, 1.01)	0.80 (-1.12, 2.72)	-0.44 (-1.95, 1.08)	-1.08 (-2.56, 0.40)
	0.0641	0.5146	0.6721	0.4152	0.5738	0.1535
Tertile 1	Reference	Reference	Reference	Reference	Reference	Reference
Tertile 2	1.11 (-0.22, 2.44) 0.1012	1.20 (-0.00, 2.41) 0.0506	0.25 (-0.93, 1.43) 0.6806	1.07 (-1.67, 3.80) 0.4461	1.79 (-0.36, 3.94) 0.1035	0.53 (-1.58, 2.64) 0.6219
Tertile 3	2.02 (0.73, 3.31) **0.0023**	1.18 (-0.01, 2.38) 0.0515	0.60 (-0.56, 1.76) 0.3119	2.66 (-0.01, 5.32) 0.0510	1.44 (-0.69, 3.57) 0.1860	0.56 (-1.52, 2.64) 0.5985
*P*_trend_	1.01 (0.37, 1.66) **0.0022**	0.60 (0.00, 1.19) **0.0490**	0.30 (-0.28, 0.88) 0.3120	1.33 (-0.00, 2.66) 0.0511	0.73 (-0.33, 1.79) 0.1790	0.28 (-0.76, 1.32) 0.5964

Crude Model = unadjusted. Model 1 = Crude Model + age, gender. Model 2 = Model 1 + race/ethnicity, education level, firearm noise exposure, occupational noise exposure, recreational noise exposure, serum cotinine, BMI, hypertension, diabetes.

### Multivariate regression analysis stratified by age: Association of 3-PBA with hearing thresholds

Table 4 shows results for 3-PBA levels in analyses stratified by age. 3-PBA levels demonstrated statistically significant interactions with age on the prediction of low-frequency and high-frequency PTA hearing threshold shifts (*P*_interaction_ = 0.0396 and 0.0017, respectively). People aged 20–39 years in the highest tertile of 3-PBA levels had higher low-frequency and high-frequency PTAs compared to those in the lowest tertile of 3-PBA levels after adjusting for age, gender, race/ethnicity, education level, BMI, hypertension, diabetes, serum cotinine level, noise exposures (β = 1.53, 95% CI: 0.04–3.01, and β = 3.14, 95% CI: 0.99–5.29, respectively) (Table 4). Gender and 3-PBA levels showed no statistically significant interactions on the prediction of hearing threshold shifts (S1 Table).

**Table 4:** Adjusted^a^ associations between 3-PBA and hearing threshold shifts stratified by age (N = 726).

	Age	Urinary 3-PBA (μg/g creatinine) β (95% CI) *P* value			*P*_trend_	*P*_interaction_
	(year)	Tertile 1	Tertile 2	Tertile 3		
**Low-frequency PTA**	20 ≤ y < 40	Reference	0.27 (-1.09, 1.62) 0.6988	1.53 (0.04, 3.01) **0.0446**	0.0503	**0.0396**
	40 ≤ y <60	Reference	0.30 (-1.85, 2.46) 0.7835	0.75 (-1.25, 2.76) 0.4628	0.4597	
	60 ≤ y <69	Reference	-1.06 (-4.89, 2.76) 0.5875	-1.07 (-4.69, 2.56) 0.5654	0.5770	
**High-frequency PTA**	20 ≤ y < 40	Reference	0.02 (-1.94, 1.97) 0.9874	3.14 (0.99, 5.29) **0.0044**	**0.0065**	**0.0017**
	40 ≤ y <60	Reference	0.49 (-3.20, 4.18) 0.7949	-0.29 (-3.72, 3.14) 0.8677	0.8548	
	60 ≤ y <69	Reference	-3.36 (-11.22, 4.51) 0.4045	-2.27 (-9.72, 5.18) 0.5514	0.5782	

^a^ Adjusted for age, gender, race/ethnicity, education level, firearm noise exposure, occupational noise exposure, recreational noise exposure, serum cotinine, BMI, hypertension, diabetes.

## Discussion

The current study examined the relationship between urinary pyrethroid metabolite 3-PBA and hearing threshold shifts of adults in the United States in a nationwide cross-sectional study. In this research, we have described a positive association between 3-PBA and both low-frequency and high-frequency PTAs in the 20–39 years old people. To the best of our knowledge, this is the first cross-sectional study that explores the relationship between individual urinary pyrethroid metabolites and hearing threshold shifts of adults in the United States. The findings indicated that pyrethroid exposure might have detrimental effects on human hearing status and that pyrethroid insecticides were prone to be more toxic to auditory system in younger adults than in older ones.

The metabolites of pyrethroid insecticides are excreted in urine which makes urine the most suitable matrix for assessing the exposure of humans to pyrethroid insecticides [25]. The detection rate of pyrethroid biomarker 3-PBA in the current research was about 92.1%, whereas only a small percentage of the other two metabolites was detectable in participants. Therefore, urinary 3-PBA was considered as a biomarker for pyrethroid exposure in our analysis. It is one of the main biomarkers used and detected in human biomonitoring [26]. The weighted mean concentration of 3-PBA was 1.44 ± 2.64 μg/g creatinine among adults aged 20–69 years old in the United States which is consistent with those reported in other studies [27,28]. The exposure of human beings to pyrethroids is worldwide.

Human studies to evaluate the effect of pyrethroids exposure on HL are few and contradictory [15,17–19,29]. Four of these studies were occupational epidemiology studies and could not avoid the influence of other insecticides on the subjects studied [17–19,29]. These data of occupationally exposed workers could not reflect the effect of pyrethroid exposure to the general people. Only one previous study reported the association of pyrethroid exposure with the risk of HL in general people [15]. The researchers found the correlation of pyrethroid exposure and HL in adolescents. The findings in our study suggested adverse effect of pyrethroid exposure in young adults, which is consistent with this study. Pregnant women, infants and children are considered groups of special vulnerability to the adverse effects of pesticides, and are of public concern [30,31]. The results of our study suggested that young adults may also be susceptible to pyrethroid exposure and needs public attention. As fruits and vegetables are the main source of exposure to pesticides in the general population, thorough washing of fruits and vegetables before eating is recommended [32]. Other measures such as reducing the spraying of pesticides and reducing the use of consumer products containing pyrethroids can be taken to reduce exposure levels of pyrethroids.

The mechanisms of increasing pyrethroid exposure to be risk for hearing is still unknown. One study showed ototoxicity of subchronic inhalation exposure to cypermethrin (type II pyrethroid pesticide) in Wistar rats by testing Distortion Product Otoacoustic Emissions (DPOAE) amplitudes [16]. DPOAE is a screening device widely used to evaluate the function of peripheral auditory system, especially outer hair cells in the cochlea [16]. Other animal studies focused on the effects of pyrethroid exposure on the nerves system showed that the toxicity of pyrethroids may through inflammation, oxidative damage, and apoptotic cell death [33]. Its main target in humans is the voltage-gated sodium channel. Chloride channels, voltage-gated calcium channels, and potassium channels are also targeted) [34]. The toxicity of pyrethroids on the auditory system may through effecting on both hair cells in the cochlea and auditory nerves.

Our study has several strengths. First, the analyses were based on a large and nationally representative sample from the NHANES. Second, pure-tone hearing threshold was measured instead of self-reported HL. Third, confounding factors including age, gender, race, education level, BMI, diabetes, hypertension, serum cotinine level, and noise exposure were adjusted in our analyses for that could result in a misinterpretation [35].

Despite these strengths, some limitations exist. First, the results of this study did not permit a cause-effect relation to be examined because the NHANES is of cross-sectional design. It will be important to continue examining these relationships in longitudinal follow-up studies. Second, we only analyzed data of people in the United States in this study. Statistical analysis of data from broad-ranging surveys in different countries, like China can provide more evidence to support the findings in our investigation. Third, though being a standard approach, pure tone audiometry was used to measure HL in this nationally representative survey, the data would be with more value if evaluation with more sophisticated methods such as DPOAE would have been added to the survey design. In addition, only main confounders reported in previous studies based on prior knowledge and the correlations between exposures and outcomes were included in calculation in the analytical models of this study [15,35–41], which cannot exclude the influence of all the other potential confounders, especially chemical influences, such as chlorinated pollutants or proximity to dioxin sources as incinerators. Furthermore, a mechanistic exploring of the effect of pyrethroid exposure on hearing impairment is lacking. More studies in the future are needed to verify these findings in this study.

## Conclusion

We observed a positive relationship between 3-PBA and both low-frequency and high-frequency hearing threshold shifts in adults aged 20–39 years old in the United States, which indicated the vulnerability of young adults to the toxicity of pyrethroid insecticides. Further studies will be required to confirm these findings.

## Supporting information

S1 Table Adjusted associations between 3-PBA and hearing threshold shifts stratified by gender (N = 726). (DOCX)
