# Supplementary material for: Urinary pyrethroid metabolite and hearing threshold shifts of adults in the United States: A cross-sectional study
Source: PLoS One. 2022 Oct 17;17(10):e0275775. doi: 10.1371/journal.pone.0275775 (PMC9576071; doi:10.1371/journal.pone.0275775)
Supplement: S1 Table — (DOCX) [file pone.0275775.s001.docx]

**S1 Table. Adjusted associations between 3-PBA and hearing threshold shifts stratified by gender (N = 726)**

|  |  | Urinary 3-PBA (μg/g creatinine) β (95% CI) *P* value | | *P* _interaction_ |
| --- | --- | --- | --- | --- |
|  |  | Male | Female |  |
| **Low-frequency PTA** | Crude Model | 1.15 (-0.20, 2.51) 0.0961 | 0.61 (-0.69, 1.90) 0.3584 | 0.5670 |
|  | Model 1 | 0.12 (-1.11, 1.35) 0.8480 | 0.58 (-0.58, 1.75) 0.3285 | 0.5912 |
|  | Model 2 | 0.16 (-1.05, 1.36) 0.8005 | 0.20 (-0.94, 1.34) 0.7292 | 0.9563 |
| **High-frequency PTA** | Crude Model | 1.89 (-0.85, 4.64) 0.1763 | 0.61 (-2.01, 3.23) 0.6483 | 0.5055 |
|  | Model 1 | -1.26 (-3.45, 0.94) 0.2621 | 0.28 (-1.80, 2.36) 0.7925 | 0.3153 |
|  | Model 2 | -1.82 (-3.97, 0.33) 0.0975 | -0.42 (-2.45, 1.60) 0.6819 | 0.3465 |

Crude Model = unadjusted. Model 1 = Crude Model + age, race/ethnicity. Model 2 = Model 1 + education level, firearm noise exposure, occupational noise exposure, recreational noise exposure, serum cotinine, BMI, hypertension, diabetes.
